# Immunotherapy for Head and Neck Cancer: A Paradigm Shift From Induction Chemotherapy to Neoadjuvant Immunotherapy

**DOI:** 10.3389/fonc.2021.727433

**Published:** 2021-09-06

**Authors:** Hirofumi Shibata, Shin Saito, Ravindra Uppaluri

**Affiliations:** ^1^Department of Medical Oncology, Dana-Farber Cancer Institute, Boston, MA, United States; ^2^Department of Otolaryngology, Gifu University Graduate School of Medicine, Gifu, Japan; ^3^Department of Otolaryngology – Head and Neck Surgery, Keio University School of Medicine, Tokyo, Japan; ^4^Department of Surgery/Otolaryngology, Brigham and Women’s Hospital, and Dana-Farber Cancer Institute, Boston, MA, United States

**Keywords:** head and neck squamous cell carcinoma, neoadjuvant immunotherapy, clinical trial, biomarker, pathological tumor response

## Abstract

Neoadjuvant immunotherapy has the potential to enhance clinical outcomes by increasing anti-tumor immune responses in the presence of abundant tumor-derived antigen in an immune microenvironment that has not been exposed to previous therapy. The current mainstay of advanced head and neck squamous cell carcinoma (HNSCC) treatment remains surgery and radiotherapy with/without conventional chemotherapy. Despite this multi-modality treatment, advanced human papillomavirus (HPV)-negative HNSCC shows poor prognosis. Treatment intensification with neoadjuvant (induction) chemotherapies with platinum drugs are insufficient to significantly prolong overall survival. Although only 15-20% of patients benefit, immunotherapies have been approved and widely used for recurrent and metastatic HNSCC. These successes have led to checkpoint blockade therapies being testing in earlier treatment settings. Recent clinical trials of neoadjuvant immunotherapy show promising results and this methodology has the potential to change the treatment algorithm of HNSCC. This overview examines the treatment history of neoadjuvant approaches for HNSCC, and especially focuses on the recent topics of neoadjuvant immunotherapy for HNSCC.

## Introduction

Squamous cell carcinoma (SCC) is the predominant malignant histology of the mucosal surfaces of the head and neck (HN) region that includes the oral cavity, pharynx, and larynx. Conventional HNSCC is mainly caused by habitual alcohol drinking and smoking, and often occurs in older adults, while human papillomavirus (HPV)-related HNSCC of the oropharyngeal region is rapidly increasing in relatively younger patients (1). The head and neck region is anatomically complex and serves essential functions such as eating, speaking, and breathing. Multi-disciplinary treatments, integrating surgery, chemotherapy, and radiation, aim to maximize treatment effects but have significant functional impact. Historically, surgery and radiotherapy with/without conventional chemotherapy including platinum, taxanes or fluorouracil, were applied to treat HNSCC. Therapeutically, HPV-positive HNSCC demonstrates sensitivity to chemoradiotherapy, and offers a better prognosis (2).

Post-operative adjuvant treatments for locally advanced HNSCC have been studied for many years as historically surgery alone for locally advanced disease had very poor outcomes. Several landmark trials established the clinical benefit of using cisplatin-based chemoradiotherapy after surgery for locally advanced, high-risk HNSCC patients (3, 4). These early studies led to two randomized Phase III trials, which provided Level 1 evidence supporting the use of concurrent chemoradiotherapy in high-risk HNSCC patients (5–7). Although these Level 1 data established a new postoperative standard of care to treat high-risk HNSCC patients, the five-year survival rate in for these patients remains suboptimal.

However, the five-year survival rate is still below 50% in advanced HPV-negative HNSCC patients (8), and many patients suffer from severe impact on essential functions. Furthermore, although distinct tumor-suppressor mutations including *TP53, CDKN2A, NOTCH* have been reported in HNSCC, cancer-promoting driver oncogenic mutations have not been detected (9–11), which makes it challenging to apply molecular targeted therapies.

Checkpoint inhibitors (CPI) targeting the programmed death 1 (PD-1) pathway have been approved for recurrent and metastatic (R/M) HNSCC patients in the first- and second-line settings (12–14) and have dramatically changed the treatment algorithm of HNSCC. The effects of checkpoint inhibitors are mainly derived from reinvigoration and activation of tumor-oriented antigen-specific T cells (15). HNSCC shows a relatively high tumor-mutational burden (TMB) (16) and immune infiltration (17), consistent with a potential to achieve therapeutic efficacy from cancer immunotherapy.

The landmark phase III CheckMate 141 trial resulted in the approval of nivolumab in the R/M second-line HNSCC setting (12). Following this, the phase III KEYNOTE-048 trial established a new paradigm for first-line R/M HNSCC patients (14). Based on this study and depending on the programmed death-ligand 1 (PD-L1) combined positive score (CPS) either pembrolizumab alone or with chemotherapy represents the first choice for these patients (14). Overall, only 15-20% of patients ultimately benefit from anti-PD-1 in these studies highlighting the need for improving efficacy of CPIs for HNSCC treatment.

These encouraging findings have led to numerous ongoing studies testing combinations to improve CPI response rates and also testing these agents in other settings. We and others have focused on the definitive surgical setting with integration of neoadjuvant immunotherapy and in this review focus on historical and current approaches. Neoadjuvant chemotherapy has a long history in HNSCC where induction chemotherapy (IC) prior to conventional platinum-based chemotherapy has been tested in numerous studies HNSCC (18). The indications for IC are limited to those with significantly advanced disease and may result in a high frequency of severe adverse events. A natural extension of this work has led several groups to test whether neoadjuvant chemotherapy prior to surgery would improve clinical outcomes. However, negative Phase III trials (19, 20) in this setting have reduced enthusiasm for these approaches. Note, there are institution specific protocols where induction chemotherapy prior to surgery is still used for larger tumors to achieve more rapid control (21). The goal of cytotoxic chemotherapy in this setting is to directly attack tumor cells to reduce tumor burden. By contrast, neoadjuvant immunotherapy is fundamentally distinct as it targets the host immune system to attack tumor cells in a durable fashion. In this review, we present a brief overview of the history of neoadjuvant (induction) chemotherapy in the definitive surgical management of HNSCC. Then, we focus on the rationale and clinical trials of neoadjuvant immunotherapy and its potential impact on HNSCC treatment.

## Induction Chemotherapy for HNSCC

In addition to the adjuvant chemotherapy, platinum-based neoadjuvant chemotherapy (induction chemotherapy; IC) has also been examined to augment subsequent (chemo)radiotherapy or surgery. The goals of induction chemotherapy are to achieve rapid tumor responses in particular with large volume disease and to “chemo-select” patients prior for definitive (chemo)radiotherapy or surgery. For larynx cancer, this approach was initially focused on reducing metastases, and preserving laryngeal function including speech and swallowing. The landmark VA Larynx study compared IC (cisplatin and fluorouracil) followed by RT versus total laryngectomy followed by RT in advanced laryngeal cancer (22). IC resulted in larynx preservation but did not contribute to improved survival. To test the sequencing of these therapies in the laryngeal cancer setting, RTOG 91-11 compared the clinical efficacy of 1) IC followed by RT, 2) CCRT and 3) RT alone for advanced laryngeal cancer patients (23). The data and subsequent meta-analysis showed the superiority of CCRT to preserve the larynx in advanced laryngeal cancer patients (8, 23).

To determine the survival benefit of IC using docetaxel plus cisplatin and fluorouracil (TPF) regimen followed by CCRT, two-phase III randomized trials were completed: the PARADIGM trial reported in 2013 (19) and DeCIDE trial reported in 2014 (20). Both trials did not show a significant extension of OS and DFS, consistent with the subsequent studies (24, 25). Importantly, phase III clinical trials which examined the clinical efficacy of IC treatment prior to surgery also failed to show suppression of loco-regional relapse and distant metastasis or extend OS (26–28). These results underscore that TPF IC is not recommended for survival benefit. In addition, IC may increase the possibility of severe AEs as compared to CCRT in non-surgical locally, advanced HNSCC treatment. However, IC remains an attractive approach for specific cases of advanced disease with a high risk for local or distant failure or to “debulk” rapidly growing tumors (19).

## Immunotherapy for R/M HNSCC

Despite these efforts to improve clinical prognosis, the five-year survival rate of locally advanced stage III/IV HNSCC patients is still sub-optimal [53% in postoperative CCRT treated patients (7)], and half of advanced patients show recurrence within three years (8). Immune checkpoint blockade therapies, especially anti-PD-1 and anti-CTLA4, were first approved in advanced melanoma patients (29) and then applied for various cancers (30), which has dramatically impacted the cancer treatment algorithm. In HNSCC, anti-PD-1 agents (nivolumab, pembrolizumab) were first examined and approved in R/M setting. The checkmate 141 phase III trial evaluated the effect of anti-PD-1 (nivolumab) for R/M HNSCC patients (12). Positive results from this study established the application of anti-PD-1 for R/M HNSCC treatment, and proved the existence of actionable, efficient anti-cancer immunity in HNSCC tumors. Similarly, the Keynote-040 randomized phase III trial compared the efficacy of pembrolizumab (anti-PD-1) versus SOC (methotrexate, docetaxel, or cetuximab) (13) for R/M HNSCC patients after platinum-containing treatment. These trials led to US Food and Drug Administration (FDA) approval of the use of anti-PD-1 (nivolumab and pembrolizumab) for second-line for recurrent and metastatic HNSCC patients who had already experienced platinum-based therapies (31).

Subsequently the Keynote-048 study, a randomized multi-center phase III study from 37 countries, examined pembrolizumab alone or with chemotherapy (platinum plus fluorouracil) versus cetuximab with chemotherapy (the EXTREME regimen (32)) for first-line treatment of R/M HNSCC (14). In this trial, pembrolizumab monotherapy significantly improved the OS of PD-L1 positive (CPS ≥20 or CPS ≥1) HNSCC. Additionally, R/M HNSCC patients treated with pembrolizumab plus chemotherapy had significantly prolonged OS compared to the cetuximab with chemotherapy group. This trial highlighted the effectiveness of combination immunotherapy and chemotherapy for subsets of HNSCC patients. Based on KEYNOTE-048, the FDA approved use of pembrolizumab monotherapy in the first-line for R/M HNSCC with CPS ≥1 and pembrolizumab plus platinum-based chemotherapy for those with CPS<1 R/M HNSCC (31).

## Rationale of Neoadjuvant Immunotherapy for HNSCC

The significant impact of checkpoint inhibitor therapy for R/M HNSCC has proven the existence of anti-cancer immunity in HNSCC (12–14). Thus, targeting immune suppression pathways with checkpoint inhibitors has been broadened to the exploration of therapeutic options in all HNSCC treatment settings. Notably, the timing of immune checkpoint inhibitors may influence the outcome of cancer treatment (33). There are now numerous studies introducing neoadjuvant immunotherapy in diverse cancer types (34–36). Considering the treatment naïve situation and the absence of treatment-resistant cells compared with the R/M setting, neoadjuvant immunotherapy is hypothetically likely able to result in a strong and durable therapeutic effect. In a spontaneous mouse metastatic breast cancer model, neoadjuvant checkpoint inhibitors showed an enhanced survival compared to the adjuvant setting by suppressing metastatic lesions (37). Intriguingly, in preclinical mouse models, a specific interval between neoadjuvant immunotherapy and subsequent surgery was important to establish potent systemic T cell response (33), suggesting that it will be important to establish the optimal duration in the clinical setting.

There are three major potential benefits to use CPIs in the neoadjuvant setting. First, neoadjuvant immunotherapies will enhance systemic T cell responses for tumor-specific antigens before surgery (34). The premise of neoadjuvant immunotherapy is to use the existing tumor mass as an in-situ source of tumor-specific antigens to enhance systemic immunity *via* dendritic cell antigen presentation to rejuvenate T cells and priming especially for cytotoxic T cells (34). This enhanced function acts to destroy micro-metastasis in clinically advanced tumors, decreasing loco-regional or distant metastasis after primary therapies. In support of this, neoadjuvant anti-PD-1 treatment in a mouse HNSCC model resulted in conversion of functional immune-dominance and induced robust anti-cancer responses, supporting the application of neoadjuvant immunotherapy for HNSCC (38). Second, in contrast to conventional chemotherapy, immunotherapy is much better tolerated by patients. Considering the high-frequency of severe adverse events and lack of significant effect OS prolongation with induction chemotherapy, neoadjuvant immunotherapy thus represents an attractive option for advanced HNSCC treatment. Finally, considering the ease of biopsies in the head and neck region, compared to adjuvant immunotherapy, neoadjuvant immunotherapy has the benefit to enable translational efforts such as TCR analysis, gene-expression profiling, and cytokine evaluation in the primary tumor which is not affected by other treatments including chemotherapeutics or radiation. These studies with previously untreated tumors may enable establishment of predictive biomarkers to select appropriate patients and also define mechanistic pathways.

## Patient Selection for Neoadjuvant Immunotherapy

An important consideration in neoadjuvant immunotherapy approaches is appropriate patient selection. Completed and ongoing trials have focused on a diverse group of HNSCC patients including early and advanced stage and HPV-positive and negative patients. This diverse patient selection has been used primarily to define a “signal” of activity. However, as immunotherapy has associated toxicities (see section on this below) and is expensive, careful patient selection to determine who may benefit from these approaches is critical. We and others have focused on HPV-negative, locally advanced disease patients with high-risk pathologic features (positive surgical margins or extra-nodal extension). These patients have the worst prognosis despite multimodality approaches and may benefit from neoadjuvant/adjuvant immunotherapy. As trials mature, patient selection for neoadjuvant immunotherapy will need to be defined further.

## Biomarker Candidates for Neoadjuvant Immunotherapy

Given that CPIs are still expensive drugs and sometimes induce severe immune-related toxicities, it is important to establish the appropriate markers which can predict efficacy of CPIs (39, 40). PD-L1 expression in tumor cells and immune cells remains the most widely used biomarker in HNSCC and other cancers (40, 41). In the KEYNOTE-048 phase III trial, significant survival benefit of pembrolizumab for patients was seen with PD-L1 expression ≥ 1% and ≥ 20% by CPS (14). In addition, in the KEYNOTE-040 phase III study, the correlation of clinical outcome and PD-L1 expression on tumor (PD-L1 tumor proportion score ≥ 50%) was evident (13). However, PD-L1 negative tumors sometimes respond to CPI treatment, suggesting the existence of other mechanisms. The expression level of PD-L1 in the tumor does not necessarily correlate with the response to CPIs. In Checkmate-141 phase III trial, there was no correlation of survival extension and PD-L1 expression on tumors (PD-L1+ >1%, 5% and 10%) (12). These data indicate that PD-L1 expression on tumor cells is not a “perfect” biomarker to predict the clinical outcome. In addition, the dynamic expression change of PD-L1 with tumor heterogeneity also makes it difficult to evaluate the expression of PD-L1 (41). Other work showed that PD-L2 expression was significantly correlated with PD-L1 expression in HNSCC clinical samples (42). Tumors with both PD-L1 and PD-L2 expression responded better than tumors with only PD-L1 expression, indicating that combinatorial scoring may be an attractive approach.

HPV infection might also be a clinical biomarker to predict the response to CPIs. HPV-related oropharyngeal HNSCC shows better survival related to HPV-negative oropharyngeal HNSCCs. HPV infection results in production of virus-related proteins, which may induce *de novo* T cell response and more CD8+ T cell infiltration in tumor (43). In the KEYNOTE-055 phase II trial, the response rate to pembrolizumab was 22% for p16 positive patients and 16% for p16 negative patients (44). A meta-analysis which examined the results of clinical trials including Checkmate 141, KEYNOTE-012, KEYNOTE-055 showed that HPV infection status was associated with the response rate to anti-PD-1 treatment independently of PD-L1 expression and TMB in HNSCC (45). Another meta-analysis showed that HPV positive HNSCC patients display significant improved outcomes with PD-1/PD-L1 axis blockage treatment compared to HPV negative HNSCC patients (46). As further investigation of these intriguing results is needed, the SITC HNSCC immunotherapy guidelines does not recommend using HPV status for anti-PD1 treatments in R/M HNSCC (31).

TMB is a potential predictive biomarker that also needs further exploration. The probability of response to CPIs has at least in part been linked to TMB across cancer types, including HNSCC (16). Patients with high-TMB have more effective clinical responses with improved survival in lung, bladder, and head and neck cancer patients (47, 48). Given that the genomic analyses of HNSCC has not identified widely shared oncogenic driver mutations but shows relatively high TMB (49, 50), the relationship between TMB and response to CPIs is promising. A study in over 300 patients across 22 solid tumor types from four KEYNOTE trials and an observational study of 126 HNSCC patients revealed HNSCC patients with high TMB showed significantly better anti-PD-1 response (51, 52). Intriguingly, TMB was significantly higher among HPV-/EBV- responders and correlated with OS, but not high in HPV+/EBV+ responders who didn’t show any correlation between TMB and OS (52). These data suggest that virus infection status impacts TMB as a biomarker. Notably, other work has contradicted the above studies on TMB and concluded that that high TMB failed to predict the effect of ICI (53). Thus, further studies are needed to define the role of TMB as a predictive biomarker.

Immune cells phenotypes in TME may also be important to predict the response to CPIs. HNSCC patients with high CD8+ T cells infiltration showed better anti-PD-1 response in the adjuvant setting (52, 54). In addition, CD8+ T cells with lymphocyte-activation gene 3 (LAG-3) or T cell immunoglobulin domain and mucin domain-3 (TIM-3) co-expression with PD-1 was higher among non-responders (52). Furthermore, tertiary lymphoid structures (TLS) in the tumor bed are suggested to contribute favorable outcome (55). Considering the TME will be dramatically changed after therapeutic treatment, neoadjuvant immunotherapy for HNSCC can provide an opportunity to establish immune markers to predict efficacy of subsequent immunotherapy.

## Pathologic Response Criteria for Neoadjuvant Immunotherapy

How to accurately evaluate the effect of neoadjuvant immunotherapy is an evolving area. For example, radiological tumor examination is widely used in Response Evaluation Criteria In Solid Tumors (RECIST) after organ preservation therapy including radiotherapy and chemotherapy. In the neoadjuvant immunotherapy context, immune-modified RECIST (imRECIST) criteria have been proposed (56). However, some immunological therapeutic effects can induce pseudo-progression or development of new lesions because of infiltration of immune cells into the primary tumor or lymph nodes, which makes it difficult to evaluate the treatment efficacy only with radiographical information (57). In fact, a study evaluating 20 resected non-small cell lung cancer (NSCLC) tumors after neoadjuvant anti-PD-1 treatment showed a discrepancy between radiological and pathological evaluation (58). These findings highlight the clinical importance to establish standard pathological criteria to accurately evaluate the therapeutic effect of neoadjuvant immunotherapy after definitive surgery.

**Figure 1:**
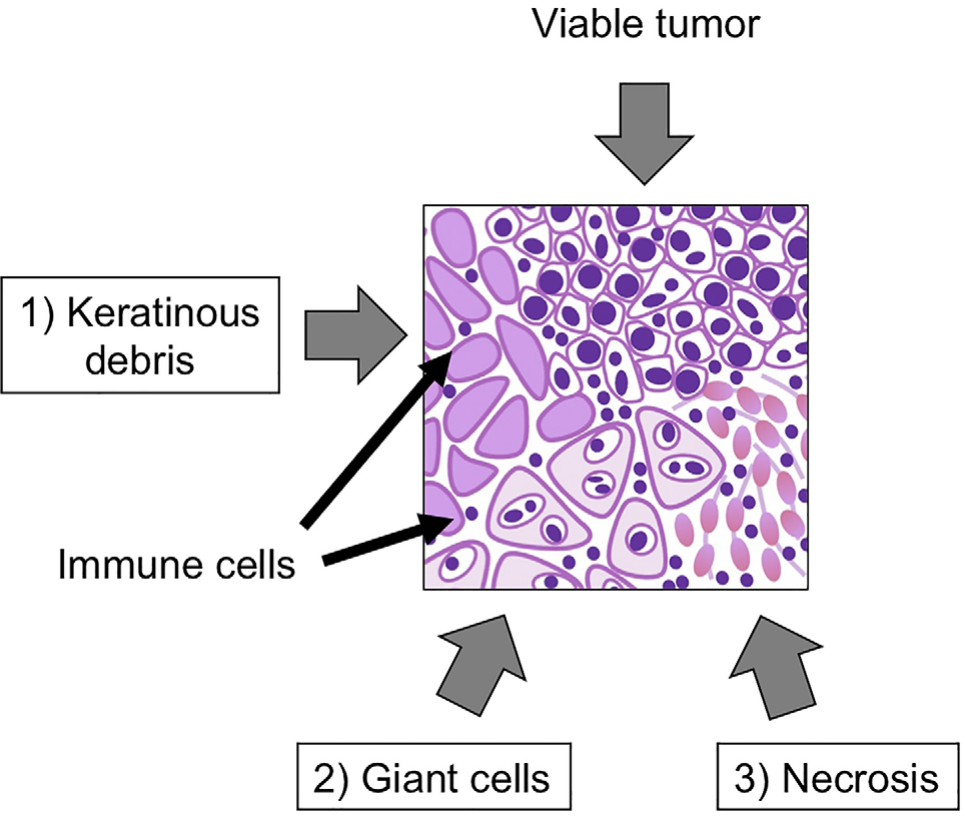
Representative figure of pathological tumor response (pTR). Key pathological findings after neoadjuvant immunotherapy include 1) keratinous debris, 2) giant cells, histiocytic reaction and 3) tumor necrosis. The pTR rate is calculated as the area of regions 1-3/(1-3)+area of any remaining tumor.

Pathological complete response (pCR) and major pathologic response (MPR) are widely used as surrogate clinical endpoints for long-term survival (59–62). Pathologic complete response means the ablation of all cancer cells in resected tumor after the treatment. On the other hand, MPR represents ≤ 10% of residual viable tumor (63). However, while pCR and MPR are considered the “gold standard”, they do not take into account lesser degrees of immunological reaction in the tumor that may still impact clinical outcomes. In fact, meta-analysis of melanoma neoadjuvant immunotherapy trials has shown that any degree of pathologic response and not just MPR/pCR, was correlated with better clinical outcomes (64). We defined pathological tumor response (pTR) as one such approach which is quantified as the proportion of the resection bed with tumor necrosis, keratinous debris, and giant cell/histiocytic reaction were distinct from growing tumor and only seen after therapy (**Figure 1**). We classified pTR into pTR-0 (≤10%), pTR-1 (≤10-49%), and pTR-2 (≥50%) (54). Pathologic treatment effect (PTE) is another similar scale, which is evaluated by the area showing fibrosis or lymphohistiocytic inflammation divided by total tumor area (65). The establishment of the best pathological method to evaluate the response of neoadjuvant immunotherapy is still evolving as the ultimate clinical impact of histologic changes is understood.

## Clinical Studies of Neoadjuvant Immunotherapy for HNSCC

With the positive responses in the R/M HNSCC setting, several trials have reported results with neoadjuvant checkpoint immunotherapy prior to surgery (**Table 1**). The phase II Checkpoint Inhibitors Assessment in Oropharynx cancer (CIAO) trial (NCT03144778) tested a combination of durvalumab (1500 mg) and tremelimumab (75 mg) in the neoadjuvant setting, preceding SOC (surgery with or without radiation therapy) (70). The primary endpoint of this trial was comparison between arms of a change in the CD8+ tumor infiltrating lymphocyte (TIL) density. A total of 28 patients were eligible, and 24 (86%) of patients were HPV positive. The combinatorial therapy group did not significantly increase the CD8+ TILs. Although neither baseline CD8+ T cell infiltration status nor PD-L1 expression level correlated with overall response, there was a trend in which greater CD8+ T cells infiltrated patients tended to show MPR. Note that MPR was observed in 8 (29%) patients in either the primary tumor or lymph node metastasis. The CD8+ T cell data was correlated with preclinical models, where anti-PD-1 and anti-CTLA4 combinatorial therapy increased tumor-infiltrating CD8+ T cells (71).

**Table 1:** Completed neoadjuvant immunotherapy clinical trials.

NCT number (Trial name)	Phase	Title	Protocol	Immunotherapy Drugs	Primary endpoint	Ref.
NCT03238365 (Merlino et al.)	I	Discordant Responses Between Primary Head and Neck Tumors and Nodal Metastases Treated With Neoadjuvant Nivolumab: Correlation of Radiographic and Pathologic Treatment Effect.	neoadjuvant	nivolumab	RVR, PTE	(65)
NCT03247712 (Leidner et al.)	Ib	Neoadjuvant immunoradiotherapy results in high rate of complete pathological response and clinical to pathological downstaging in locally advanced head and neck squamous cell carcinoma.	neoadjuvant/adjuvant	nivolumab	surgical delay, pCR, MPR, pathological downstaging	(66)
NCT02488759 (Checkmate 358)	I/II	Neoadjuvant nivolumab for patients with resectable HPV-positive and HPV-negative squamous cell carcinomas of the head and neck in the CheckMate 358 trial.	neoadjuvant	Nivolumab	safety and tolerability, response rate, surgical delay	(67)
NCT02919683 (Schoenfeld et al.)	II	Neoadjuvant Nivolumab or Nivolumab Plus Ipilimumab in Untreated Oral Cavity Squamous Cell Carcinoma: A Phase 2 Open-Label Randomized Clinical Trial.	neoadjuvant	nivolumab, ipilimumab	Safety, volumetric response	(68)
NCT02296684 (Uppaluri et al.)	II	Neoadjuvant and Adjuvant Pembrolizumab in Resectable Locally Advanced, Human Papillomavirus-Unrelated Head and Neck Cancer: A Multicenter, Phase II Trial.	neoadjuvant/adjuvant	pembrolizumab	safety, pTR-2,1-year relapse rate	(54)
NCT03021993 (Xiong et al.)	II	Immunological effects of nivolumab immunotherapy in patients with oral cavity squamous cell carcinoma.	neoadjuvant	nivolumab	pathological response	(69)
NCT03144778 (Ferrarotto et al.)	II	Impact of Neoadjuvant Durvalumab with or without Tremelimumab on CD8(+) Tumor Lymphocyte Density, Safety, and Efficacy in Patients with Oropharynx Cancer: CIAO Trial Results.	neoadjuvant	durvalumab, tremelimumab	CD8+ TILs density	(70)

RVR, Radiographic volumetric response; PTE, Pathologic treatment effect; pCR, pathological complete response; MPR, major pathological response; pTR, pathological tumor response.

Schoenfeld et al. examined neoadjuvant 1) nivolumab (N) or 2) nivolumab plus ipilimumab (N+I) in untreated 29 oral cavity cancer patients in a phase II trial (eligible for ≥ T2 or node positive) (NCT02919683) (68). Nivolumab (3 mg/kg) was administered on weeks 1 and 3, while ipilimumab (1 mg/kg) was given on week 1 only. Although a total of 21 patients experienced AEs, including grade 3/4 AEs in 2 (N) and 5 (N+I) patients, there were no surgical delays. In addition, there was evidence of response in both arms. Notably, four patients (N, n=1; N+I, n=3) had major/complete response (greater than 90%). These data suggest clinical tolerability and effectiveness of neoadjuvant immunotherapy.

We reported a phase II trial, in which neoadjuvant/adjuvant pembrolizumab was tested in locally advanced, resectable HPV-negative HNSCC patients (NCT02296684) (54). In this trial, safety, pTR, and relapse rate with pembrolizumab were evaluated. A total of 36 patients (T3/T4; 80%, stage IV; 92%) were enrolled and received one time dose of neoadjuvant pembrolizumab (200 mg) followed by surgery two or three weeks after the immunotherapy. Per standard of care, postoperative RT or CCRT were performed, and adjuvant pembrolizumab treatment was used in high-risk patients with positive surgical margins or extra-nodal extension. Notably, grade 3/4 serious adverse events or delay of surgery didn’t occur, underscoring the safety of neoadjuvant immunotherapy. Furthermore, the one-year relapse rate in high-risk patients was 16.7%, which was lower than historical data. The pTR scores were evaluated by two independent pathologists and graded using the following scale: pTR-0 < 10%, pTR-1; 10-49%, pTR-2 ≥ 50%. Any pTR was seen in 44% and pTR-2 was seen in 22% of patients. Notably, any pTR after neoadjuvant pembrolizumab correlated with baseline tumor PD-L1, immune infiltration, and IFN-*γ* activity, but not TMB. These data suggest the reactivity of neoadjuvant immunotherapy is related to immunogenic phenotype before treatment and highlights the future possibility to select patients for neoadjuvant immunotherapy before surgery.

Merlino et al. reported on findings from a clinical trial where neoadjuvant nivolumab (240 mg on days 1 and 15) with or without tadalafil was tested. Patients received two cycles of drug therapy. The radiographic volumetric response (RVR) and PTE were evaluated, and the results of RVR and PTE was significantly correlated in primary tumor and lymph nodes. Intriguing findings from this study reported discordant responses between primary tumor and regional metastatic lymph nodes (NCT03238365) (65).

A phase II trial was reported by Xiong et al. (NCT03021993), in which a total of 10 locally advanced OSCC patients were treated with neoadjuvant nivolumab (3 mg/kg on days 1, 14 and 28) (69). The immunological responses were analyzed using blood before and after treatment. Although this study didn’t report pathologic responses or clinical efficacy, the proportion of CD8+ T cells, especially granzyme B positive cells, increased after treatment. However, the proportion of CD4+ T cells were decreased while the rate of CD4+FoxP3+ regulatory T cells was increased with treatment. These data highlight the difficulty of interpreting peripheral lymphocyte populations with clinical responses in HNSCC patients treated with neoadjuvant immunotherapy.

The Checkmate 358 phase I/II study examined clinical safety and efficacy of two doses of neoadjuvant nivolumab in HPV positive or negative HNSCC (NCT02488759) (67). No new safety signals were observed and there were no surgical delays. Pathologic responses were evaluated in 34 patients (17 HPV+ and 17 HPV-negative). Major pathological responses were seen in 1 HPV-positive tumor with none in the HPV-negative tumors. Three HPV-positive tumors and one HPV-negative tumor had partial pathologic responses.

## Ongoing Clinical Trials

In addition to the published studies above, several ongoing neoadjuvant immunotherapy trials with subsequent surgery for locally advanced HNSCC have reported results at major oncology meetings (**Table 2**).

**Table 2:** Ongoing neoadjuvant immunotherapy clinical trials.

NCT Number (Trial Name)	Phase	Protocol	Drugs	Primary Endpoint	Ref.
NCT03238365	I	neoadjuvant	Nivolumab, Tadalafil	immune cell polarization (Th1/Th2; M1/M2)	
NCT03003637 (IMCISION)	IB/II	neoadjuvant	Nivolumab, Ipilimumab	tolerability, pathological response, hypoxia	(72)
NCT03174275	II	adjuvant/neoadjuvant	Duravalumab, carboplatin, nab-paclitaxel	pCR rate	
NCT03721757 (NICO)	II	adjuvant/neoadjuvant	Nivolumab	DFS (12 months following surgery)	
NCT03107182 (OPTIMA-II)	II	neoadjuvant	Nivolumab, Nab-paclitaxcel, Carboplatin, 5-FU, Paclitaxcel	tumor shrinkage rate with DRR	
NCT03341936	II	neoadjuvant	Nivolumab, Lirilumab	DFS	(73)
NCT03342911	II	neoadjuvant	Nivolumab, Paclitaxcel, Carboplatin	pCR	
NCT03708224	II	neoadjuvant	Atezolizumab, Tiragolumab, Tocilizumab	CD3+ T cells increase rate (≥ 40%)	
NCT03944915 (DEPEND)	II	neoadjuvant	Nivolumab	DRR	
NCT02641093	II	adjuvant/neoadjuvant	Pembrolizumab, Cisplatin	safety and benefit of adding Pembro to SOC	(74, 75)
NCT04080804	II	Neoadjuvant	Nivolumab, Relatlimab, Ipilimumab	safety, AEs rate	
NCT03765918 (Keynote-689)	III	adjuvant/neoadjuvant	Pembrolizumab, Cisplatin	MPR, event free survival (EFS)	(74, 75)
NCT03700905 (IMSTAR-HN)	III	adjuvant/neoadjuvant	Nivolumab, Ipilimumab	DFS (approximately 71 months)	

IMCISION, immunomodulation by the combination of ipilimumab and nivolumab in neoadjuvant to surgery in advanced or recurrent head and neck carcinoma; NICO, Neoadjuvant and adjuvant nivolumab as immune checkpoint inhibition in oral cavity cancer; OPTIMA-II, Chemotherapy and locoregional therapy trial for patients with head and neck cancer; DEPEND, De-escalation therapy for human Papillomavirus negative disease; IMSTAR-HN, Study of Nivolumab alone or in combination with Ipilimumab as immunotherapy vs standard follow-up in surgical resectable HNSCC after adjuvant therapy; pCR, pathological complete response; DFS, disease free survival; DRR, deep response rate; AE, adverse event; MPR, major pathological response; EFS, event free survival; PFS, progression free survival.

Updated results of a phase II neoadjuvant pembrolizumab trial prior to surgery followed by adjuvant concurrent pembrolizumab and radiation along with cisplatin for clinically high-risk (T3/4 stage and/or ≥ 2+ LNs) HPV-negative HNSCC patients (NCT02641093) were recently presented (74). This is multi-institutional trial enrolled 92 patients and 76 patients were evaluable for DFS. They used pathological response (PR) criteria which was defined tumor necrosis and/or histiocytic inflammation and giant cell reaction to keratinaceous debris (74). Of eighty evaluated patients, 32 patients (40%) showed a PR [26 partial PR (≥ 20% and <90%) and 6 with major PR (>90%)]. Notably, patients with PR (partial plus major) showed significantly improved 1-year DFS compared to patients with no PR (100% versus 68%, p = 0.01; HR = 0.23). These are the first clear data in HNSCC supporting the finding that neoadjuvant anti-PD1 induced PR is a predictor of clinical outcomes.

The IMCISION study (NCT03003637) presented at ESMO 2020 is examining neoadjuvant nivolumab and ipilimumab for stage II-IVa HNSCC patients. This trial included both definitive and salvage surgery patients. Notably, the treatments were safe and 16/26 patients (61.5%) had pathologic responses (>20%) and 8/26 (31%) of patients experienced complete response (72).

As opposed to the CIAO and IMCISION trials where some patients enrolled were undergoing salvage surgery, a third trial recently presented at ASCO 2021 focused exclusively on challenging recurrent, surgically resectable HNSCC patients (NCT03341936) (73). Twenty-nine HNSCC patients with locoregionally recurrent disease who were surgically resectable were treated with neoadjuvant nivolumab and lirilumab, an anti-KIR blocking antibody focused on NK cell checkpoint inhibition. Patients also received 6 months of adjuvant nivolumab and lirilumab. There were no delays to surgery and 3/28 patients had Grade 3 AEs. Pathologic responses were seen in 12/28 (43%) of patients with 4 having MPR. Clinical outcomes were better than historical with 70% 1-year disease free survival and 85% 1-year overall survival.

In addition to ongoing Phase II trials, KEYNOTE-689 is an international phase III study (NCT03765918) where surgically resectable locally advanced HPV-negative HNSCC patients are randomized to receive upfront surgery with SOC adjuvant treatment or neoadjuvant pembrolizumab (two doses) followed by surgery and SOC adjuvant treatment with pembrolizumab (76). This trial aims to enroll 600 patients. In this trial, primary endpoints are rate of major pathological response (≤10% tumor cells in resected primary and lymph nodes on central review) and event-free survival (EFS). Secondary endpoints are OS, complete pathological response, and assessment of safety and tolerability.

Finally, we recently reported a second cohort of our neoadjuvant pembrolizumab trial where instead of one dose, patients received two doses of drug similar to the neoadjuvant phase of the KEYNOTE-689 Phase II trial (75). Compared to our initial cohort with one dose, we found that 50% of patients had any pTR and 44% of patients exhibited pTR2. This was nearly double what we saw with one dose of pembrolizumab. These data show that two doses or the longer neoadjuvant window (3 versus 6 weeks) resulted in an increased rate of pTR but did not increase the total proportion of patients with pTR.

## Immune Related Adverse Events in Neoadjuvant Immunotherapy Treated Patients

An important consideration in neoadjuvant immunotherapy approaches is clinical safety as the possibility of lifelong autoimmune complications in the definitive surgical setting needs to be weighed carefully. As mentioned above, to date neoadjuvant immunotherapy has been shown to be safe and has not resulted in surgical delays. In a phase II neoadjuvant immunotherapy clinical trial for oral cavity cancer patients which treated with nivolumab (N, n=14) or nivolumab and ipilimumab (N+I, n=15), two (N) and five (N+I) patients showed grade 3/4 AEs. These included oral mucositis and one patient with autoimmune diabetes (68) and there were no surgical delays. In another phase II neoadjuvant pembrolizumab clinical trial, we reported no severe grade 3/4 AEs and no surgical delays in a total of 36 treated HNSCC patients (54). Recently we reported an extension of this study with an additional 29 HNSCC patients treated with two cycles of neoadjuvant pembrolizumab. In this trial, only one patient showed a grade III AE (rash) while no patients had grade IV AE, consistent with the safety and tolerability of neoadjuvant immunotherapy (75).

## Conclusion and Future Perspectives

The published and ongoing trials described above focused on single agent checkpoint blockade immunotherapy prior to surgery. In addition to this design, immunotherapy is being integrated in several neoadjuvant combinations with radiation or chemotherapy prior to surgery. The Neoadjuvant Immuno-RadioTherapy (NIRT) phase Ib trial tested neoadjuvant stereotactic body radiation therapy (SBRT) with nivolumab (240 mg, q2 weeks x 3) prior to surgery in HNSCC patients (NCT03247712) (66). There were no treatment related delays thus achieving the primary safety endpoint. There was an 86% MPR rate and a 67% pCR rate. For all cohorts, there was a 90% clinical to pathologic down staging. NIRT did impact healing of wounds that all ultimately resolved. There were excellent clinical outcomes and only one patient required adjuvant chemoradiation. There are several questions about how this approach would integrate with current SOC including whether this treatment intensification is necessary especially in good prognosis HPV+ disease and the role of nivolumab as SBRT alone conferred a high rate of pathologic responses. In addition to radiation and immunotherapy combinations, other trials are testing chemotherapy/immunotherapy combinations. For example, in a phase II trial, platinum combined with immunotherapy (nivolumab) followed by transoral robotic surgery (TORS) or RT/CRT is being examined in oropharyngeal cancer patients (NCT03107182). Using a primary radiation based approach, several ongoing clinical trials aim to de-intensify the treatment impact by adding immunotherapy (77). For example, a phase II/III trial in patients with early-stage HPV-positive HNSCC is testing whether RT plus chemotherapy (cisplatin) or immunotherapy (nivolumab or durvalumab) can be used for de-intensification (NCT03952585, NCT03410615). There are several distinct mechanisms of how radiation and/or chemotherapy can work with immunotherapy and other have covered these topics. These trials will test the important topic of whether there is synergy in combination approaches with RT, immunotherapy and/or chemotherapy.

In conclusion, we provided here an overview of the history of neoadjuvant immunotherapies in HNSCC starting with chemotherapy extending to exciting frontiers using immunotherapy. IC continues to be used at some centers with defined indications including advanced or borderline resectable tumors. Management of toxicities in this setting remains a challenge. However, although IC may help with surgical management, Phase III trial results showed no improvements in survival. It has become clear that neoadjuvant immunotherapy, especially checkpoint inhibitors, are safe and have shown signals of clinical efficacy in HNSCC. These data together support further investigation in Phase III trials such as KEYNOTE-689 to define evidence for survival benefit and identify high-risk patients who may benefit from this approach. In addition, as other checkpoints are testing, further improvements in pathologic responses and clinical outcomes are expected. In conclusion, neoadjuvant approaches provide a potential exciting new treatment paradigm for HNSCC patients.

## Author Contributions

HS: writing original draft, tables, and figure. SS: editing the manuscript. RU: editing and supervising the manuscript, tables and figure. All authors contributed to the article and approved the submitted version.

## Funding

RU is funded by NIH/NIDCR R01DE024403, R01DE027736, and NIH/NCI/NIDCR U01DE029188. HS received funding from the Uehara Foundation (201941070).

## Conflict of Interest

RU serves on an advisory board for Merck, Inc.

The remaining authors declare that the research was conducted in the absence of any commercial or financial relationships that could be construed as a potential conflict of interest.

## Publisher’s Note

All claims expressed in this article are solely those of the authors and do not necessarily represent those of their affiliated organizations, or those of the publisher, the editors and the reviewers. Any product that may be evaluated in this article, or claim that may be made by its manufacturer, is not guaranteed or endorsed by the publisher.
